# Anthracnose Disease Control and Postharvest Quality of Hass Avocado Stored in Biobased PLA/PBAT/Pine Essential Oil/Chitosan Active Packaging Nets

**DOI:** 10.3390/plants11172278

**Published:** 2022-08-31

**Authors:** Zormy N. Correa-Pacheco, Rosa I. Ventura-Aguilar, Leonor Zavaleta-Avejar, Laura L. Barrera-Necha, Mónica Hernández-López, Silvia Bautista-Baños

**Affiliations:** 1 Centro de Desarrollo de Productos Bióticos, Instituto Politécnico Nacional, Carretera Yautepec-Jojutla, km 6, Calle CEPROBI, No. 8, San Isidro 62731, Yautepec, Morelos, Mexico; 2 CONACYT-Centro de Desarrollo de Productos Bióticos, Instituto Politécnico Nacional, Carretera Yautepec-Jojutla, km 6, Calle CEPROBI, No. 8, San Isidro 62731, Yautepec, Morelos, Mexico; 3 Tecnológico Nacional de México/I. T. Zacatepec, División de Estudios de Posgrado e Investigación, Calzada Tecnológico 27, Zacatepec de Hidalgo 62780, Morelos, Mexico

**Keywords:** *Persea americana* L., *Colletotrichum gloeosporioides*, ripening, *Rhizopus stolonifer*, fruit quality

## Abstract

For sale in the domestic market, Hass avocados are kept in non-biodegradable plastic nets and stored at an ambient temperature. The fungus *Colletotrichum gloeosporioides* can cause important losses at this stage. Consequently, formulations based on polylactic acid (PLA)/poly (butylene adipate-co-terephthalate) (PBAT) 60/40 biodegradable blends added with pine essential oil (PEO) at 10, 12, 14, and 20% and coated with 1% chitosan (CH) were used for the elaboration of nets, which were evaluated on *C. gloeosporioides* and *Rhizopus stolonifer* on nutrient media and fruit and on the ripening behavior of Hass avocados at ambient temperature. The spore germination stage of *C. gloeosporioides* was the most extensively damaged (78% inhibition). The incidence of anthracnose was notably reduced by almost 80% in the avocados stored in the 60/40 PLA/PBAT nets coated with 1% CH. The overall values regarding weight loss, °Brix, and dry matter were c.a. 23.5%, 7.5, and 24.5%, respectively. The changes in firmness, color, and CO_2_ production were particularly associated with the initial harvest index and storage temperature rather than with the nets. In future research, essays on nets should include evaluations at the commercial levels.

## 1. Introduction

The avocado (*Persea americana* L.) fruit is a species native to Mexico and Central America and an important agricultural product for human consumption worldwide due to its content of phenols, fiber, and lipids, including phytosterols, terpenoids, and unsaturated fatty acids [1].

The final postharvest handling of these fruit for national destinations involves packaging in nets and cardboard cartons and storage at ambient temperature. However, during the retail and commercialization stages, fungal diseases such as anthracnose and black mold, caused by the fungi *Colletotrichum gloeosporioides* and *Rhizopus stolonifer*, respectively, limit their marketing potential [2,3,4]. 

On the other hand, the accumulation in the environment of conventional synthetic polymers for use as containers for agricultural packaging, such as, among others, PET (polyethylene terephthalate) and HDPE (polyethylene terephthalate) obtained from fossil/petrochemical sources, has generated a large amount of plastic waste in the environment that does not degrade over long periods, causing severe pollution problems [5]. 

For this reason, a great effort has been made to produce new biodegradable materials from renewable sources for manufacturing agricultural containers. These materials include PLA (polylactic acid), which can be used as a packaging material due to its biodegradability in carbon dioxide, water, and other molecules under composting conditions [6]. Equally, the fully biodegradable PBAT (butylene adipate-co-terephthalate), marketed under common trade names such as Ecoflex Wango, Ecoworld, Eastar Bio, and Origo-Bi, is a flexible material with good hydrophilic properties and compatible with PLA [7].

Another important natural polymer is chitosan, which is used in the development of functional packaging materials due to, among other factors, its non-toxic properties, biocompatibility, biodegradability, and fungicidal properties concerning plants and various horticultural products during storage [8,9]. In addition, this polymer can be added to different formulations that contain PLA as mentioned above and PBAT polyesters [6].

On the other hand, essential oils extracted from different parts of plants are complex mixtures of secondary metabolites. Among other applications, these compounds have shown notable results as antimicrobials on various fungi such as, among others, *Alternaria alternata*, *C. gloeosporioides*, *Fusarium oxysporum*, and *Penicillium* spp., which are present during the storage of horticultural products [10]. Therefore, this biological activity deserves to be considered and evaluated for its inclusion in biodegradable containers. 

Pine essential oil is commonly obtained from flowers, leaves, and seeds from coniferous trees of the genus *Pinus*, including the species *P. sylvestris* (common name: Scots pine). Concerning its biological activity, diverse studies have pointed out that essential oils from *Pinus*, such as *P. halepensis*, *P. mugo*, *P. muricata*, *P. nigra*, *P. ponderosa*, *P. radiata*, and *P. sylvestris,* among others, with nematicidal (pinewood nematode), bactericidal (food-borne pathogens), fungicidal (*Aspergillus* spp., *Fusarium* spp.), insecticidal (*Drosophila melanogaster*), and herbicidal activities (*Sinapis arvensis*, *Phalaris paradoxa*, and *Raphanus raphanistrum*), primarily associate this characteristic with the secretion of an oleoresin consisting of turpentine (monoterpenes and sesquiterpenes) and rosin fractions (diterpenes) [11,12,13,14,15,16].

On this subject, relevant results were obtained by Segura-Palacios et al. [17] and Ventura-Aguilar et al. [18] with a nanocoating-based pine resin extract (commercial name: Resindeher) + propolis + chitosan. The authors highlighted their inhibitory effect on the fungus *A. flavus*, in vitro, on tomatoes and tortillas (a food derivative of maize). 

According to Restuccia et al. [19] an active packaging is defined as ‘the incorporation of certain compounds into packaging systems to extend the shelf life, maintain or improve the food quality, being their main purpose to release or absorb those compounds into or from the packaged food or the environment surrounding the food’, with the advantage that a diversity of active agents such as, among others, enzymes, fungicides, plant extracts, and essential oils, can be incorporated into the packaging.

Generally, for postharvest uses, polymeric materials are used as raw materials to produce packaging materials, such as agricultural product containers in the form of plastic nets. Castellano et al. [20] defined them as ‘threads connected together with each other, in a woven or knitted way forming a regular porous geometric structure and allowing gases to go through’. To date, the use of plastic nets as packaging containers is rapidly increasing. However, this has become a matter of concern since they are manufactured with materials, including HDPE, PE (polyethylene), and PP (polypropylene), which are petroleum-based polymers.

In line with this subject, Black-Solís et al. [21] tested the effect of biodegradable nets manufactured from extruded fiber-based PL/PBAT/cinnamon bark essential oil on the ripening of tomatoes and the incidence of *A. alternata*. The results showed no side effects on cherry tomato quality during storage and an incidence of this fungus that was slightly lower in those tomatoes previously grown under greenhouse conditions in the biodegradable nets compared to the fruit grown without them.

The objectives of this research were then to evaluate an active packaging in the form of net-based PLA/PBAT/pine essential oil/chitosan on (1) the in vitro growth of *C. gloeosporioides* and *R. stolonifer* and (2) the anthracnose disease development and ripening behavior of Hass avocado fruit, stored at ambient temperature in commercial and non-commercial nets, for a given storage period of nine days.

## 2. Results and Discussion

Table 1 details the 13 major compounds of *P. sylvestris* out of 26. Among those that were identified are isoterpinolene, terpinolene, and eucalyptol, which are classified within the groups of monoterpenes and terpenes.

This information agrees with that previously reported by Rodrigues et al. [15] and Sjödin et al. [22]. They identified the following in the oil of this same species: monoterpene hydrocarbons, sesquiterpene hydrocarbons, and oxygenated mono and sesquiterpenes, ranging from 50% to 90%.

In other studies, in the case of Macedonian pine leaves, 90 compounds were found, the dominant ones being α-pinene (45.5%), germacrene D (11.1%), β-pinene (10.8%), and camphene (10.3%), corroborating that the terpenes group is the majority found in pine species [23].

In the in vitro evaluations, for both fungi, the fungicidal activity was manifested mainly in 2 of the 5 treatments that contained *P. sylvestris* oil, i.e., T4 (60/40 PLA/PBAT + 12% PEO) and T8 (60/40 PLA/PBAT + 20% PEO + 1% CH). 

Regarding the mycelial growth, *C. gloeosporioides* was the most affected by T8 but without any damage to *R. stolonifer* (Table 2). Contrary to these results, Motiejūnaitė and Pečiulytė [13] reported the MIC90 values in *R. stolonifer* to range from 0.75 to 0.5. This is probably due to the evaluation methodology since these authors assayed the agar diffusion method. Other phytopathogenic fungi that responded to the fungicidal effect of the *P. sylvestris* essential oil were *A. alternata*, *A. flavus*, *A. niger*, *A. versicolor*, *Cladosporium cladosporioides*, *F. oxysporum*, and *Phoma* spp. [24].

**Figure 1:**
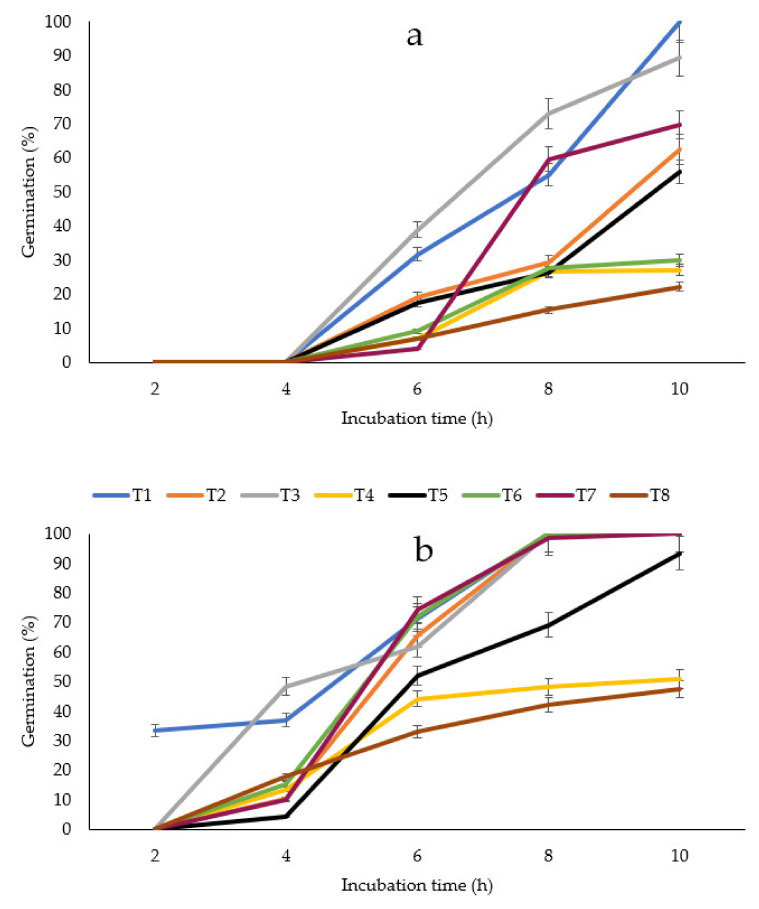
Conidial germination of (**a**) *Colletotrichum gloeosporioides* and (**b**) *Rhizopus stolonifer* incubated in the presence of nets manufactured with 60/40 PLA/PPBAT + PEO at different concentrations and 1% chitosan for 10 h at 20 °C. Bars indicate the standard error of the mean. (See Table 4 for treatments description).

As for the germination of spores, the formulations T4 and T8 also affected this growing stage in the two tested fungi. For example, in *C. gloeosporioides* incubated in the presence of these treatments, the spore inhibition was approximately 78% (Figure 1a), while for *R. stolonifer,* it was about 55% (Figure 1b).

In line with this research, Black-Solis et al. [21] also tested the effect of nets elaborated with PLA/PBA nets + cinnamon essential oil coated with chitosan on *A. alternata* growth, resulting in a notable fungicidal activity on mycelial growth with recently manufactured nets and those which were 15 days old. Furthermore, except for the control treatment, the nets with 6.1% cinnamon essential oil and coated with 2% and 3% chitosan fully inhibited the spore germination of this fungus. 

**Figure 2:**
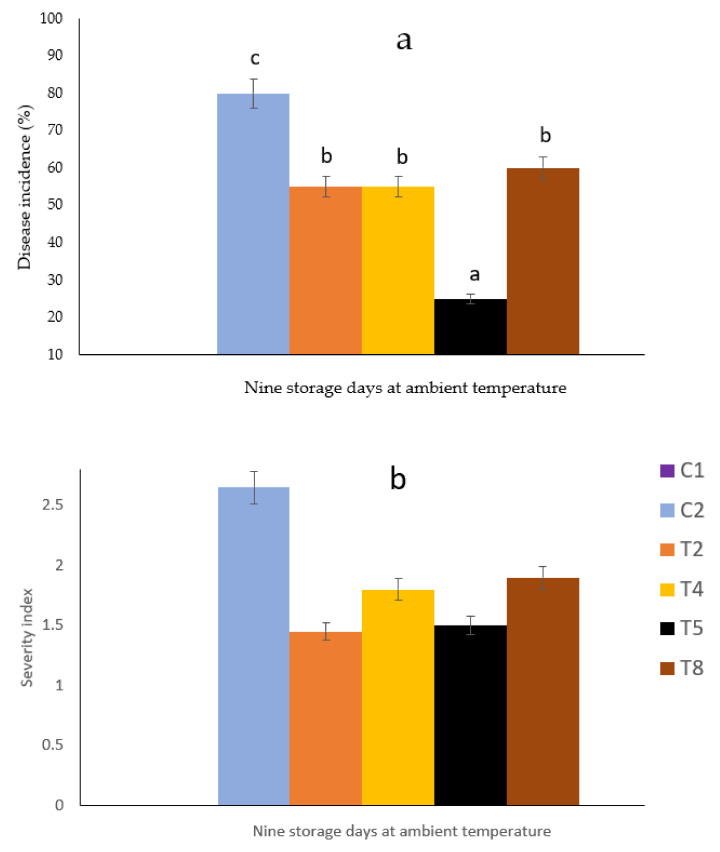
Disease incidence (**a**) and severity index (**b**) of Hass avocado stored in nets manufactured with 60/40 PLA/PPBAT + PEO at different concentrations + 1% chitosan for nine days at ambient temperature. ^a, b, c^ Different letters mean significant differences (*p* < 0.05; Tukey test) among treatments. Bars indicate the standard error of the mean. The severity index 2 = 1–25% of the fruit surface being rotten. Control treatments in unbagged avocados in nets: C1 = non-inoculated and C2 = inoculated fruit. In inoculated and bagged avocados in nets: T2 = 60/40 PLA/PBAT, T4 = 60/40 PLA/PBAT + 12% PEO, T5 = 60/40 PLA/PBAT + 1% CH, and T8 = 60/40 PLA/PBAT + 20% PEO + 1% CH.

Concerning the disease incidence variable (Figure 2a), the highest control (of approximately 80% of anthracnose disease) was shown in avocados kept in nets of T5 (60/40 PLA/PBAT + 1% CH), followed by 45% of disease control in T2 (60/40 PLA/PBAT) and T4 (60/40 PLA/PBAT + 12% PEO). The incidence of anthracnose with regard to the treatment T8 (60/40 PLA/PBAT + 20% PEO + 1% CH) and control inoculation was higher than 50%. The fruit surface with anthracnose symptoms was less than 25% in T2 and T5 (Figure 2b).

In this study, it was clear that there was not a defined pattern concerning the net composition and the control of anthracnose. Perhaps, in this case, the disease control was more associated with the intrinsic defense mechanisms of avocado fruit against pathogenic microorganisms, as discussed by Herrera-González et al. [25]. These authors highlighted that once a fungal pathogen attacks avocado fruit, a chain of processes is activated, such as, among others, secondary metabolite production, structural responses, defense and antioxidant enzymatic reactions, and an array of gene expressions.

For the avocados ripening after the nine-day storage period, it can be seen in Table 3 that only the variables firmness, color, and CO_2_ production were significantly different (*p* < 0.05) among the various treatments. In this case, the greatest firmness (of about 40 N) and the highest changes in the skin (ΔE 9.8) and mesocarp color (ΔE 15.5) corresponded to the fruit kept in T5 (60/40 PLA/PBAT + 1% CH) and T8 (60/40 PLA/PBAT + 20% PEO + 1% CH) nets, while the lowest CO_2_ production (38.0 mg CO_2_ kg^−1^ h^−1^) was in those held in T2 (60/40 PLA/PBAT) nets. 

As mentioned earlier, using nets based on PLA/PBAT/PEO with chitosan kept the avocado fruit slightly firmer. In agreement with these results, Zhang et al. [26] reported similar outcomes concerning passion fruit. In that research, the polymeric package based on PLA/PBAT kept the fruit firmer than the polyethylene packaging. However, in our experiments, the avocados’ rapid loss of firmness could be more due to the stored temperature of 25 ± 2 °C for nine consecutive days. In this respect, Zauberbam and Jobin [27] and Marquez et al. [28] found a high correlation between storage temperatures higher than 22 °C and the final low firmness of Hass avocados.

Overall, the blackening of the fruit skin of the avocados in this experiment could be particularly associated with the initial harvest index, the normal ripening process, and perhaps damage caused by *C. gloeosporioides*. According to Cox et al. [29], the skin of Hass avocados color generally changes from green to purple/black as the fruit ripens due to an initial decrease in the content of chlorophyll α and β, followed by an increase in the content of anthocyanin cyanidin 3-O-glucoside’. In our experiments, the overall change of color of the avocado skin from green to purple and black in the given nine-day storage period at ambient temperature agreed with the color associated with the optimal maturity of this commodity.

Generally, in our research, from harvest until the end of the nine days of storage, the use of polymeric nets, with or without PEO, did not deeply influence the production of CO*_2_* in the fruit. Overall, the range in the matter of treatments was from 40.1 to 42.0 mg CO_2_ kg^−1^ h^−1^. In previous studies carried out on tomatoes also kept in nets based on PBA/PBAT with and without cinnamon essential oil, it was discussed that, overall, their respiratory rate increased after harvest, but those tomatoes not stored in nets and from nets with cinnamon essential oil had the highest respiration rate, which was also seen in our study [21].

In our research, the final average weight loss values among treatments were c.a. 23.5%, °Brix, 7.5, and dry matter, 24.5%, which fell within the values reported in other studies. 

In the case of weight loss, lower values from 6.9% to 11.4% were reported by Herrera-González et al. (2017) [30]. These results were likely due to the storage temperature since their reported storage temperature was 22 ± 1 °C, while ours was 25 ± 2 °C.

For °Brix, Márquez et al. [28] marked an average value of 4.7 for the late maturity state of avocadoes, while Astudillo-Ordóñez and Rodríguez [31] reported a °Brix range from 5.0 to 7.2, which agreed with the results we obtained.

In the case of the variable dry matter, the values at harvest were very similar to those we obtained. In this respect, the dry matter did not increase during storage, as others have reported [28,30,32]. These authors generally associated the dry matter with the fruit oil content, harvest period area, and environmental conditions.

## 3. Materials and Methods

### 3.1. Materials and Manufacturing of Nets

The materials used to manufacture the fibers were pellets of 60% Polylactic acid (PLA) (Ingeo TM Biopolymer 7001D from NatureWorks LLC., Minnetonka, MN, USA), 40% Poly (butylene adipate-*co*-ter- ephthalate) (PBAT) (Ecoflex F Blend C1200, from BASF (Mexico City, Mexico), 10, 12, 14, and 20% pine essential oil (*Pinus sylvestris* L.) (PEO) bought from Xipe Naturals (Mexico City, Mexico), and 1% commercial chitosan (CH) obtained from America Alimentos Ingredientes Funcionales (Mexico City, Mexico). 

The nets were made following the methodology of Hernández-López et al. [33]. The polymer blend (60% PLA and 40% PBAT) was dried at 60 °C for 24 h. A total of 30 g of this mixture was processed in a twin screw mini extruder (Thermo Scientific, Process 11. Santa Clara, CA, USA). The fibers of 60/40 PLA/PBAT involving pine essential oil were elaborated by adding it through a peristaltic pump, which changed speed from 0.10–0.20 mL/min. The fibers were cooled in a water bath at the end of the extrusion. In the case of the chitosan-coated fibers, this compound was used instead of water. 

### 3.2. Chemical Characterization of the Pine Essential Oils Using GC-MS 

Chromatograph analysis of the pine essential oil was carried out according to the methodology proposed by Black-Solis et al. [34]. The pine essential oil was diluted in chloroform (1:10 *v*/*v*). Then, 1 μL of the dissolution was injected into a gas chromatograph (GC) SCION 456- GC (Bruker Daltonics, Inc., Billerica, MA, USA) adapted to an EVOQ mass triple quadrupole detector and a PAL-COMBI automatic sample injector (Bruker Daltonics, Inc., Billerica, MA, USA). The mass spectrometer (MS) was set to a 50 to 500 *m*/*z* ratio range using the MS Workstation version 8.2 software. The GC-MS was equipped with a BR-1ms capillary column 30 m long, 0.25 mm in internal diameter and 0.25 μm in thickness (Bruker Daltonics, Inc., Billerica, MA, USA). The injector and detector temperatures were 220 °C and 280 °C, respectively. Helium (He) was used as carrier gas at a 1 mL min^−1^ flow rate. The initial oven temperature was 55 °C for 1 min and was then increased to 155 °C at a heat speed rate of 20 °C min^−1^. This was maintained for 2 min and then increased to 255 °C at a heat speed rate of 10 °C min^−1^ for a total analysis period of 20.14 min per sample. The detected compounds were identified by comparing their retention time and mass spectrum with data from standards included in the NIST (National Institute of Standards and Technology, Gaithersburg, MD, USA) team’s library. 

### 3.3. Fungicidal Activity on Hass Avocados

#### 3.3.1. Fungal Isolates

The strains of *C. gloeosporioides* and *R. stolonifer* were obtained from the fungi collection of the Postharvest Technology Laboratory of Agricultural Products of CEPROBI (Morelos, Mexico). The fungi were grown in a potato dextrose agar (PDA) nutrient culture (Bioxon, Mexico City, Mexico) for 15 and 5 days, respectively, at 25 °C. 

#### 3.3.2. In Vitro Studies

##### Evaluation of Mycelial Growth, Inhibition Index, and Spore Germination 

**Figure 3:**
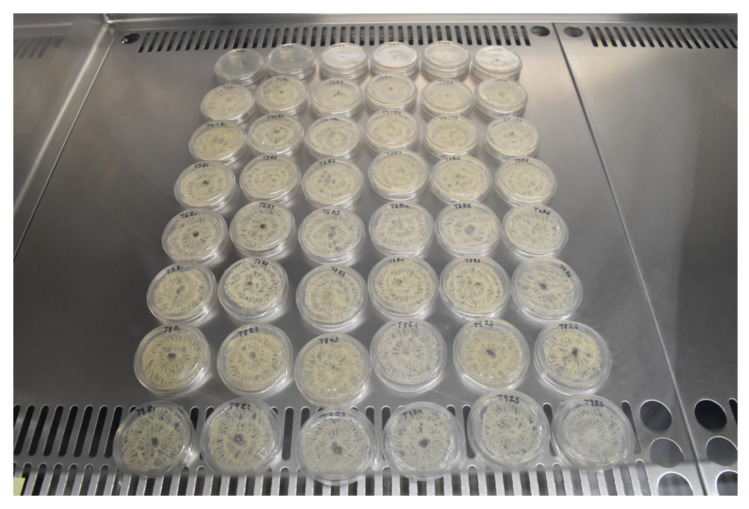
Petri plates containing PDA nutrient agar and net-based PLA/PBA/pine essential oil/chitosan.

The antifungal effect was evaluated in terms of mycelial growth, inhibition index, and percentage spore germination following the methodology of Black-Solis et al. [21] with some modifications. For mycelial growth and growth inhibition, an agar disk of 5 mm in diameter approximately from actively growing cultures of *C. gloeosporioides* and *R. stolonifer* was placed in the center of a Petri plate (60 × 15 mm^2^) with PDA. Fibers of approximately 60 cm in diameter (1.15 g of weight) were then placed on the plate’s lid, sealed, placed upside down and incubated at 25 °C (Figure 3). The control plate consisted of growing each isolate only in PDA. The volumetric concentration of the CEO in the fiber was calculated using the average diameter of the fiber (d) and the total fiber length (LF) in cm. The radial growth measurements were taken from six plates for each fungus at 13 and seven days, respectively.

For the variable germination, the fungal spores from the treatments of three Petri plates were harvested by scraping them off the agar with a glass rod and distilled water. The resulting filtrate was adjusted to 10^5^ spores/mL. Twenty µl of this spore solution was placed on 15 mm PDA agar disks. Three inoculated disks were placed separately on a glass slide (12 mm × 10 mm), kept in empty Petri plates, sealed, and incubated at 20 °C for two, four, six, eight, and 10 h. At the end of the incubation periods, the number of germinated conidia was counted with an optical microscope (NIKON, Osaka, Japan) at 40×. 

The treatments for assays are shown in Table 4.

#### 3.3.3. In Situ Studies

##### Treatment Application

**Figure 4:**
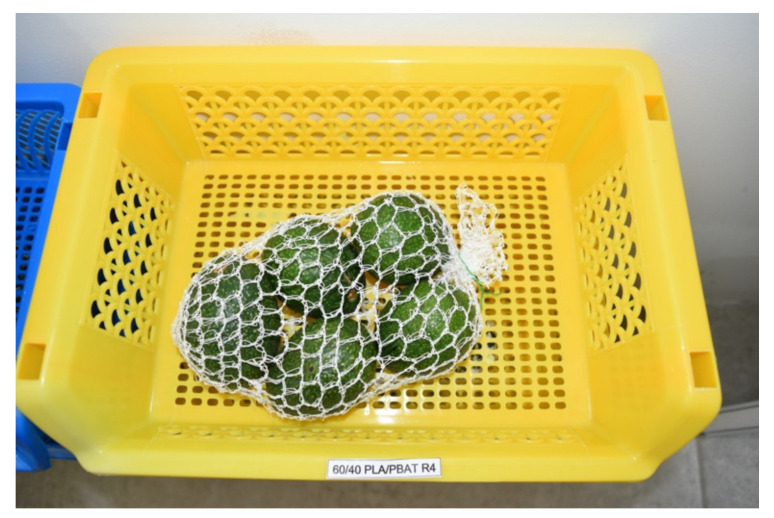
Avocado fruit bagged in net-based 60/40 PLA/PBA/pine essential oil at different concentrations + 1% chitosan and stored at 25 ± 2 °C for nine days.

The Hass avocados fruit were harvested from an orchard located in the Tlalnepantla municipality, state of Morelos. The harvested fruit followed the Mexican Norm NMX-FF-016-SCFI-2016 [35], i.e., grade class II (complete fruit, free of rots, foreign materials, insects or pests, any strong odor and taste, and dry material less than 21%). The fruit weight was approximately 115 to 150 g. They were washed with a solution of distilled water for 15 min, followed by a second immersion in 1% sodium hypochlorite for 15 min, and rinsed again with sterile distilled water. In the fruit, two wounds of 2 mm depth were made, and they were inoculated with 10 μL of a spore solution of *C. gloeosporioides* of 10^5^ spores mL^−1^. The fruit was left to dry and bagged in the nets (five fruit per net). The avocados were then stored at room temperature (25 °C ± 2 °C) for nine days (Figure 4). 

The final treatments were control treatments in the case of unbagged avocados: C1 = non-inoculated and C2 = inoculated fruit. The avocados inoculated and bagged in nets were: T2 = 60/40 PLA/PBAT, T4 = 60/40 PLA/PBAT + 12% PEO, T5 = 60/40 PLA/PBAT + 1% CH, and T8 = 60/40 PLA/PBAT + 20% PEO + 1% CH.

##### Disease Incidence and Severity Index of Hass Avocados

The variable disease incidence was evaluated as a percentage of infected avocados. Small portions of the symptomatic tissue were placed on Petri plates containing PDA and incubated for 15 days at 25 °C to verify *C. gloeosporioides* on the samples. Conidia were identified using optical microscopy at 40X [36]. With respect to the variable severity index, it was ranked 1 to 5, where 1 = 0%, 2 = 1–25%, 3 = 26–50%, 4 = 51–75%, and 5 = 76–100% of the surface of fruit with symptoms of anthracnose disease. The two variables were evaluated in 5 fruit per treatment and 4 replicates.

### 3.4. Quality and Ripening of Hass Avocados

#### Weight Loss, Firmness, TSS, Dry Matter, Color, and CO_2_ Production

Ripening of the bagged and non-bagged fruit was evaluated according to the following variables: weight loss, firmness, color, CO_2_ production [37], TSS [38], and dry matter [39]. The fruit was stored for nine days at 25 ± 2 °C. All variables were measured at the end of this storage period with 10 fruit per treatment and 4 replicates.

Weight loss was determined as the difference between each avocado’s initial and final weight using a digital balance (OHAUS CS2000. Sigma Aldrich. Mexico City, Mexico). Data were expressed as percentages. 

A penetrometer with an 8.0 mm probe (Fruit Firmness Tester TR 53205, Turoni Forli, Italy) was used for firmness. Measurements were taken at two opposite sides of the fruit, and the averaged values were reported as Newtons (N). 

The total soluble solids (TSS) were determined with an Atago N-1E refractometer (USA Inc. Bellevue, WA, USA) and expressed as °Brix. For this, five grams of the avocado pulp were mixed in distilled water and homogenized at 2500 rpm for 15 min. A drop of the mixture was placed in the refractometer, and values were recorded. 

The dry matter was determined by the technique of drying to constant weight with a convection drying oven. For this, the seed and avocado peel were removed, and longitudinal slices of fruit approximately 2 mm thick were cut to obtain 10 g. The samples were then placed on previously weighed brown paper sheets (10 × 13 cm^2^) and dried in a convection drying oven (BINDER ED 53-UL. Sigma Aldrich. San Luis, MO, USA) until a constant weight was achieved. The dry matter was calculated using the equation: DM (%) = (M1 (sample initial weight)/M0 (sample final weight)) × 100. The variable of color was evaluated in the fruit epidermis and mesocarp in two equatorial points of the avocado’s middle region with a Konica Minolta Colorimeter (Baking Meter BC-10, Konica Minolta Sensing, Osaka, Japan). Values of L*, a* and b* were averaged and expressed as a color change (ΔE) using the following equation: ΔΕ = [(L_2_ − L_1_)^2^ + (b_2_ − b_1_)^2^ + (a_2_ − a_1_)^2^]^1/2^.

For the CO_2_ content, the head-space technique was used. This consisted of recovering 0.5 mL of gas contained in 2 L glass jars in which the avocados were kept for 1 h. The gas samples were injected into a gas chromatograph model 7890B (Agilent Technologies, Santa Clara, CA, USA) supplied with HP-PLOT/Q and CP-Molsieve 5A columns (Agilent Technologies, Santa Clara, CA, USA). The carrier gas was helium at a flow rate of 10 mL min^−1^. The injector was used in split mode (1:10), and the temperatures of the injector and detectors FID and TCD were 220, 250, and 300 °C, respectively. The retention time was 2.6 min. The respiratory rate was expressed as mg CO_2_ kg^−1^ h^−1^.

### 3.5. Statistical Analysis

Treatments were arranged in a completely randomized design. Means and standard error of the mean were also calculated. Data were analyzed using ANOVA, and mean comparisons were carried out using the Tukey test at *p* < 0.05. 

## 4. Conclusions

In terms of the planned objectives, it is concluded that polymeric nets’ in vitro fungicidal effect occurred mostly in *C. gloeosporioides*. In this case, the spores are the most affected stage of the fungus development, with a germination inhibition of 78% when incubated in the presence of 60/40 PLA/PBAT + 20% PEO + 1% CH nets. In addition, the incidence of anthracnose was notably deterred by almost 80% in avocados stored in the nets based on 60/40 PLA/PBAT + 1% chitosan. The ripening process of the avocado fruit was not affected by using any of the tested polymeric nets, i.e., they continued to ripen normally during the 9 days storage period at ambient temperature. 

## Figures and Tables

**Table 1:** Chemical profile of the main compounds of *Pinus sylvestris*.

Compound Identified	Retention Time (min)
1.-(R) α pinene	6.335
2.-Camphene	6.623
3.-1,4-Cieneole	8.142
4.-p-Cimene	8.322
5.-Eucalyptol	8.538
6.-c–Terpinene	9.307
7.-Terpinolene	10.099
8.-Fenchol	10.604
9.-α-Terpineol	11.146
10.-b-terpineol	11.364
11.-endo-Borneol	11.952
12.-Terpineol	12.595
13.-Isoterpinolene	12.794

**Table 2:** Growth of *Colletotrichum gloeosporioides* and *Rhizopus stolonifer* in the presence of polymeric nets manufactured with 60/40 PLA/PPBAT + PEO at different concentrations + 1% chitosan for 10 h at 20 °C.

Treatments	*Colletotrichum gloeosporioides*		*Rhizopus stolonifer*	
	Mycelial Growth (cm)	Growth Inhibition (%)	Mycelial Growth (mm)	Growth Inhibition (%)
T1	53.0 ± 0 ^a^	0.0	53.0 ± 0 ^a^	0.0
T2	53.0 ± 0 ^a^	0.0	53.0 ± 0 ^a^	0.0
T3	51.5 ± 2.4 ^ab^	2.8	53.0 ± 0 ^a^	0.0
T4	48.2 ± 1.3 ^ab^	9.0	53.0 ± 0 ^a^	0.0
T5	52.0 ± 2.3 ^a^	1.8	53.0 ± 0 ^a^	0.0
T6	52.0 ± 2.3 ^a^	1.8	53.0 ± 0 ^a^	0.0
T7	46.0 ± 2.1 ^b^	12.3	53.0 ± 0 ^a^	0.0
T8	43.1 ± 2.2 ^c^	19.0	53.0 ± 0 ^a^	0.0

^a, ab, b, c^ Different letters mean significant differences (*p* < 0.05; Tukey test) among treatments. Means ± standard error of the mean were calculated. (See Table 4 for treatments description).

**Table 3:** The effect of polymeric nets manufactured with 60/40 PLA/PPBAT + PEO at different concentrations + 1% chitosan on the final maturity of Hass avocado fruit after nine days of storage at ambient temperature.

Treatments	Weight Loss (%)	Firmness (N)	TSS (°Brix)	Dry Matter (%)	Color (ΔE)		CO_2_ Production (mg CO_2_ kg^−1^ h^−1^)
					Epidermis	Mesocarp	
C1	22.5 ^a^	21.3 ^b^	7.8 ^a^	21.7 ^a^	4.4 ^a^	11.8 ^ab^	41.1 ^c^
C2	23.6 ^a^	20.5 ^b^	7.1 ^a^	23.2 ^a^	5.3 ^a^	9.8 ^a^	41.1 ^c^
T2	23.4 ^a^	14.6 ^a^	8.1 ^a^	24.3 ^a^	8.3 ^b^	9.9 ^a^	38.0 ^a^
T4	23.8 ^a^	35.9 ^c^	7.5 ^a^	24.4 ^a^	5.9 ^a^	10.8 ^ab^	42.0 ^c^
T5	23.6 ^a^	39.6 ^c^	7.5 ^a^	24.6 ^a^	9.8 ^b^	13.1 ^ab^	40.1 ^b^
T8	23.0 ^a^	39.0 ^c^	7.7 ^a^	23.5 ^a^	9.8 ^b^	15.5 ^b^	42.0 ^c^

Initial values of firmness = 178.1 N; °Brix = 8.5; dry weight = 23.2%; CO_2_ production = 30.1 mg CO_2_ kg^−1^ h^−1^. ^a, ab, b, c^ Different letters mean significant differences (*p* < 0.05; Tukey test) among treatments. Control treatments in unbagged avocados in nets: C1 = non-inoculated and C2 = inoculated fruit. In inoculated and bagged avocados in nets: T2 = 60/40 PLA/PBAT, T4 = 60/40 PLA/PBAT + 12% PEO, T5 = 60/40 PLA/PBAT + 1% CH, and T8 = 60/40 PLA/PBAT + 20% PEO + 1% CH.

**Table 4:** Formulations for the manufacturing of polymeric nets for in vitro and in situ treatments.

Treatments	Net Composition
T1	Control (PDA)
T2	60/40 PLA/PBAT
T3	60/40 PLA/PBAT + 10 % PEO
T4	60/40 PLA/PBAT + 12 % PEO
T5	60/40 PLA/PBAT + 1% CH
T6	60/40 PLA/PBAT + 10 % PEO + 1% CH
T7	60/40 PLA/PBAT + 14 % PEO + 1% CH
T8	60/40 PLA/PBAT + 20 % PEO + 1% CH

PDA = Agar potato dextrose; CH = chitosan; PLA = Poly lactic acid; PBTA = Poly (butylene adipate-co-terephthalate); PEO = pine essential oil.

## Data Availability

Not applicable.
